# Taking a Stand: A Prospective Study on the Influence of Posture on Urodynamic Studies in Older Patients

**DOI:** 10.3390/medicina61091576

**Published:** 2025-08-31

**Authors:** Andries Van Huele, George Bou Kheir, Alan Wein, Veerle Decalf, Thomas F. Monaghan, François Hervé, Karel Everaert

**Affiliations:** 1 Department of Urology, AZ Alma, 9900 Eeklo, Belgium; 2 Department of Urology, Ghent University Hospital, 9000 Ghent, Belgium; 3 Department of Urology, Desai Sethi Urology Institute, University of Miami Health System, Miami, FL 33136, USA; 4 Department of Urology, University of Texas Southwestern Medical Center, Dallas, TX 75390, USA

**Keywords:** lower urinary tract symptoms, urinary incontinence, urinary incontinence, urge, urinary incontinence, stress, posture, adult, frail older, home, old age

## Abstract

*Background and Objectives*: Urinary incontinence (UI) is a prevalent issue among older adults and may require urodynamic studies (UDSs) for accurate diagnosis. However, these procedures can be uncomfortable and time-consuming, especially in a geriatric population, where certain practical restrictions may apply. This study examines whether posture of filling cystometry during UDSs in an older patient group affects diagnostic outcomes and whether a single UDS in one posture is sufficient for a reliable diagnosis or if multiple postures provide added value. *Materials and Methods*: This is a secondary analysis of the Think Dry: Optimalisation of Diagnostic Process of Urinary Incontinence in Older People study (NCT04094753), a prospective observational cohort study. Each patient underwent both sitting and standing filling cystometry during UDS. The final diagnosis was determined by the referring urologist by integrating results from both the sitting and standing groups alongside all available clinical data. Subsequently, each separate UDS was reviewed independently by a second, blinded, urologist, and a diagnosis was established based on a single UDS. The agreement between these independent diagnoses and the final diagnosis was then evaluated using Cohen’s kappa coefficient (κ). *Results*: Results from the UDS with the standing filling cystometry had an almost perfect agreement (κ = 0.92) with the final diagnosis, compared to only a moderate agreement (κ = 0.42) while sitting. *Conclusions*: UDS with standing filling cystometry may be sufficient for an accurate diagnosis, potentially eliminating the need for additional filling cystometry in the sitting position. By streamlining the diagnostic process, this approach could enhance efficiency, reduce patient burden, and optimize resource utilization in older adults.

## 1. Introduction

Urinary incontinence (UI) is a prevalent condition among older adults, significantly impacting quality of life [1,2]. Urodynamic studies (UDSs), more specifically, multi-channel pressure subtracted cystometries, are the gold standard to assess voiding function and to quantify dysfunction and help to provide or confirm diagnosis, predict treatment outcome, or facilitate discussion during counseling [3,4].

The International Continence Society (ICS), supported by the Society of Urodynamics, Female Pelvic Medicine and Urogenital Reconstruction (SUFU), provides recommendations for good practices in UDSs [3]. One of the elements discussed in these standards is the importance of repeating UDSs in specific situations, particularly when abnormal bladder function is observed, discrepancies exist between medical history and expected urodynamic findings, or technical errors and artifacts are identified during immediate post-test analysis [3,5]. By adhering to these guidelines, the accuracy of urodynamic assessments can be improved, leading to more reliable diagnostic outcomes. However, clinical practices in urodynamics often differ significantly, with multiple practitioners performing two subsequent UDSs by default due to longstanding clinical habits or institutional protocols.

Another consideration in regard to UDSs is the patient’s posture during filling cystometry. The ICS-SUFU standards recommend that patients be allowed to undergo testing in their preferred position [3]. In the 7th International Consultation on Incontinence (ICI) report, based on the ICI-ICS consultation held in 2021, the committee recommends performing UDSs in the upright position (not supine) [6]. Previous studies have reported inconsistent variations in UDS parameters regarding the impact of posture on urodynamic parameters (in general, supine is less representative) and diagnostic outcomes [7,8,9,10].

Unfortunately, most of these data and recommendations are not specified for an older patient group, in which minimizing procedural burden is essential for comfort and quality of life. As repeated urodynamic tests may be fatiguing and time-consuming, identifying the most diagnostically effective posture from the outset is particularly relevant to optimize both patient experience and procedural efficiency [11]. Data in this subpopulation remain limited.

Our objective was to evaluate whether filling cystometry during UDSs performed in different postures—sitting versus standing—affects diagnostic accuracy in older patients. This study is a secondary analysis of Think Dry: Optimalisation of Diagnostic Process of Urinary Incontinence in Older People (NCT04094753), a prospective observational cohort study designed to optimize UI diagnosis in older adults. We aimed to determine whether a single UDS in one posture is sufficient for a reliable diagnosis or if performing UDSs in multiple postures provides additional value. We assessed the agreement between individual UDS results (filling cystometries in different postures) and the final diagnosis, which incorporates both UDS findings and all available clinical data. As a secondary objective, we compared specific urodynamic parameters between the sitting and standing UDSs.

## 2. Materials and Methods

### 2.1. Study Design

This study is a secondary analysis of the Think Dry: Optimalisation of Diagnostic Process of Urinary Incontinence in Older People study (NCT04094753), a prospective observational cohort study designed to optimize UI diagnosis in older adults.

### 2.2. Participants

Patients aged ≥ 65 years with any type of UI were eligible for inclusion. Exclusion criteria included the presence of an indwelling urinary catheter or patients performing clean intermittent catheterization (CIC). Of 180 patients in the database, 102 underwent both sitting followed by standing filling cystometry and were included in this analysis.

### 2.3. Procedure

Each patient underwent two UDSs or pressure-subtracted multi-channel cytometries (Aquarius CTS system, Laborie Medical Technologies, Toronto, ON, Canada), one with the filling cystometry in a sitting position, followed by one in a standing position. The voiding phase was conducted in a seated position for both groups. Urodynamic parameters were recorded according to the ICS guidelines and terminology [5,12]. The final diagnosis (stress urinary incontinence (SUI), urgency urinary incontinence (UUI), or mixed urinary incontinence (MUI); no patients had overflow urinary incontinence) was established by the referring urologist, who considered both sitting and standing filling cystometry results along with additional clinical data, including medical history, physical examination, voiding diaries, and patient-reported questionnaires.

### 2.4. Concordance

**Figure 1:**
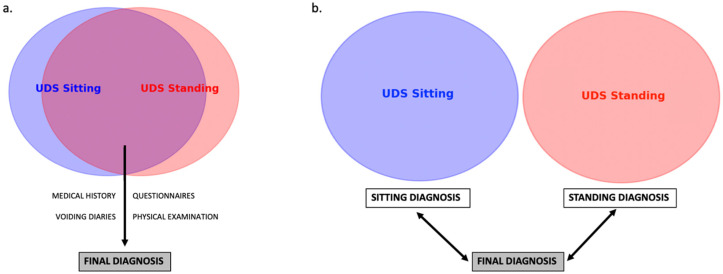
Schematic overview of methods. (**a**). Diagnostic flowchart with both UDS and clinical parameters leading to final diagnosis by referring urologist. (**b**). Independent analysis of both UDS by a second blinded urologist, with comparison—concordance analysis—to the final diagnosis (made by the referring urologist. UDS: Urodynamic study.

To assess concordance, a second urologist, blinded to clinical data and final diagnoses, retrospectively reviewed the original UDS traces. Seated and standing studies were assessed separately, without simultaneous comparison of traces from the same patient during the evaluation. Concordance between these individual UDS-based diagnoses and the original comprehensive diagnoses was then analyzed. A schematic overview is shown in Figure 1.

### 2.5. Statistical Analysis

Demographic variables were compared using Kruskal–Wallis and chi-square or Fisher’s exact tests. Urodynamic parameters were compared between sitting and standing positions using Wilcoxon signed-rank tests. Concordance between the diagnoses of the separate UDS findings and the final diagnosis was evaluated using Cohen’s kappa coefficient (κ), which categorizes agreement levels as follows: 0.00–0.20 indicates slight agreement, 0.21–0.40 represents fair agreement, 0.41–0.60 corresponds to moderate agreement, 0.61–0.80 reflects substantial agreement, and 0.81–1.00 signifies almost perfect agreement [13]. Statistical analyses were conducted using SPSS version 29.0. Graphics were created using SPSS version 29.0, R-studio, and Biorender.

## 3. Results

### 3.1. Patient Characteristics

The median age of participants was 74 years (IQR: 70–78), with 90.2% of the study population being female. UI subtypes were distributed as follows: SUI 40.2%, UUI 30.4%, and MUI 29.4%. No patients had overflow urinary incontinence. There were no significant differences between the diagnostic groups in regards to demographics, living situation, education, comorbidities, and basic clinical parameters, as seen in Table 1.

### 3.2. Individual Urodynamic Parameters

Table 2 presents the comparative analysis of the individual UDS parameters between sitting and standing positions.

The analysis found that the post-void residual volume (PVR) was significantly lower in the standing position group for the general study population (median 50 mL vs. 40 mL, *p* = 0.026). In the SUI group, bladder compliance was significantly higher in the standing position compared to the sitting position (median 94.0 mL/cmH_2_O vs. 47.7 mL/cmH_2_O, *p* = 0.026). Other urodynamic parameters, including bladder sensation thresholds, maximum and average urinary flow rates, and detrusor pressure at maximum flow, did not show significant differences. Their subgroup analysis by incontinence type yielded similar non-significant findings.

### 3.3. Concordance Analysis

**Figure 2:**
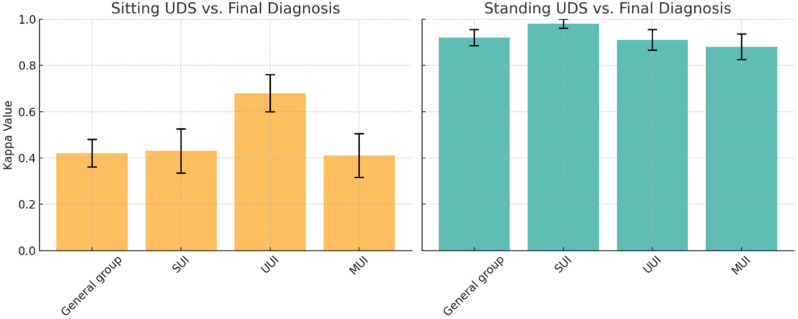
Concordance analysis (Kappa Value and SE) comparing position and urodynamic diagnosis. (**Left**): Sitting vs. final diagnosis; (**Right**): Standing vs. final diagnosis. UDS: Urodynamic study, SUI: Stress urinary incontinence, UUI: Urgency urinary incontinence, MUI: Mixed urinary incontinence, SE: Standard error.

This analysis demonstrated a moderate agreement (κ = 0.42, SE 0.36–0.48) for sitting UDSs but an almost perfect agreement (κ = 0.92, SE 0.89–0.96) for standing UDSs compared to the final diagnosis. Similar patterns were observed in subgroup analyses stratified by incontinence type (see Figure 2), with the highest kappa value being 0.98 (SE 0.96–1.00) in the standing group with the diagnosis of SUI.

## 4. Discussion

This is the first study to evaluate whether UDSs performed with different filling cystometry postures—sitting or standing—affect diagnostic accuracy in older patients. Pressure-subtracted multi-channel cystometry plays a crucial role in assessing urinary tract function, and it remains the gold standard in the diagnostic process for UI [3]. However, it is an invasive procedure associated with a reported morbidity rate up to 19.0%, including urinary retention, gross hematuria, urinary tract infections, and fever [14]. It is crucial to carefully weigh the diagnostic benefits against these risks in an older patient group. As multiple guidelines suggest, pressure-subtracted multi-channel cystometry should be reserved for cases where it leads to better outcomes and more effective clinical care for all patient groups and ages [4,15,16]. Excessive testing can increase anxiety, discomfort, and healthcare costs, emphasizing the need for a judicious approach, particularly in older populations [17,18].

Many external factors can influence the results of UDSs—such as the detection rate of detrusor overactivity (DO)—including fluid temperature, bladder filling rate, patient posture, repeated testing, and potential irritation caused by the catheter [7,10,19]. The ultimate goal of our study was to simplify and streamline the diagnostic process for older patients with UI in regards to the optimal position of the patient, possibly resulting in the reduction of repeated tests.

Our concordance analysis, which assessed the agreement between individual UDSs performed in different positions with the final diagnosis, based on both UDSs and all available clinical data, revealed an almost perfect agreement for the filling cystometry in the standing position, whereas the agreement was noticeably lower in the sitting position. Although only a single urologist reviewed the cystometry tracings in this study, prior research has demonstrated high inter-rater reliability in the interpretation of adult cystometry tracings [20]. Subgroup analysis showed that patients diagnosed with SUI in the standing position had the highest agreement compared to other subgroups and other positions. This aligns with clinical practice, where a stress cough test is preferably performed in a standing position to maximize sensitivity [21]. Taken together, since current clinical practice often involves performing two consecutive UDSs despite the absence of guideline recommendations, the “standing only” approach demonstrated an almost perfect agreement with the final diagnosis, suggesting that it could be an acceptable sole method, which would result in potential cost savings, reduced patient discomfort, and improved workflow in urodynamic units.

Our study identified several statistically significant differences in urodynamic parameters between the sitting and standing UDS groups, focusing specifically on how these parameters from the two test positions differ from each other. However, the clinical relevance of these differences should be interpreted with caution. For PVR in the general study population, the absolute difference was minimal and unlikely to be clinically significant. In the SUI group, bladder compliance in the standing position was nearly twice as high as in the sitting position (94.0 vs. 47.7 mL/cmH_2_O). However, since the ICS guidelines define compliance above 40 mL/cmH2O as normal in women, this statistically significant difference may not translate into clinical relevance [3,5].

Our findings align with the general trend in previous research emphasizing the influence of posture during filling cystometry, although there is no real consensus. Additionally, most studies primarily focus on comparing supine versus erect—sitting or standing—positions, with no direct comparisons between the latter two. Prior studies, such as the review by Al Hayek et al. and the prospective study of Arunkalaivanan et al., have demonstrated that filling cystometry performed in the supine position may fail to detect a significant proportion of DO or stress-induced leakage compared to an erect position [7,8]. The randomized controlled trial (RCT) by Jeon et al. examined both posture and repetition effects and showed that supine posture and repeated filling cystometry reduced bladder sensitivity and DO while increasing bladder compliance and maximum cystometric capacity. Interestingly, the supine posture correlated more strongly with overactive bladder (OAB) symptoms than the erect posture during the first filling cystometry [10]. The systematic review and meta-analysis by de Jong et al. found no significant differences in urodynamic parameters among healthy individuals across positions. However, in men with lower urinary tract symptoms (LUTS), sitting was associated with an improved urodynamic profile compared to standing [9]. All these data align with the 7th ICI report, in which the committee recommends performing UDSs in the upright position (not supine). It is important to note that these data do not specifically pertain to older or frail patients.

In frail patients, streamlining UDSs is particularly crucial because these individuals have increased vulnerability to invasive procedures, and little evidence shows that extensive UDSs improve their outcomes [22]. A holistic approach is desired, integrating geriatric assessment to identify reversible factors and prioritizing conservative management before resorting to invasive diagnostics and treatments [23]. However, in select cases, particularly when non-invasive assessments are inconclusive, UDSs may offer valuable insights, as UI in this patient group often arises from a complex interplay of different pathophysiological components, physiological changes, comorbidities, medications, and functional impairments [18]. Despite the importance of accurate diagnostics, research on effective treatments for frail older adults also remains limited [23]. Without evidence-based therapeutic options, even the most precise diagnostic tools have little clinical impact, highlighting the urgent need for both improved diagnostic strategies and targeted treatment approaches in this vulnerable population.

This analysis is the first to our knowledge to perform a concordance analysis and direct comparison between sitting and standing filling cystometry in UDSs exclusively in an older patient population. The large sample size strengthens statistical power and enhances generalizability. Its prospective design ensures systematic data collection, minimizing bias. The findings have direct clinical relevance and may contribute to optimizing the diagnostic flowchart for UI in older adults.

However, certain limitations should be acknowledged. The observational design limits causal inferences, and the testing sequence was not randomized, introducing a potential order effect that was not controlled for, potentially influencing the observed kappa coefficient. Future randomized trials are needed to confirm these findings and further refine clinical guidelines. Finally, while some differences were observed, it is unclear whether these would change a clinical diagnosis, prevent an operation, or lead to a different surgical approach. Urodynamic studies are not, and have never been, exact or fully repeatable investigations, and this inherent variability should be considered when interpreting the findings.

## 5. Conclusions

Standing UDSs provide very high diagnostic accuracy compared to sitting, demonstrating an almost perfect agreement with the final diagnosis. This approach may reduce the need for additional test positions beyond standing, enhancing the efficiency of the diagnostic flowchart and patient comfort. Further research, incorporating randomized testing sequences, is recommended to validate these results and guide clinical practice.

## Figures and Tables

**Table 1:** Demographics divided by diagnosis.

Demographics	All Patients (n = 102)	SUI (n = 41)	UUI (n = 31)	MUI (n = 30)	*p*-Value
Age, years (median, IQR)	74 (70–78)	73 (70–77.5)	74 (69–77)	76 (71–80)	0.36
Gender, Female (%)	92 (90.2)	36 (87.8)	28 (90.3)	28 (93.3)	0.74
**Living situation and education**					
Living together with partner, n (%)	66 (64.7)	25 (61.0)	20 (64.5)	21 (70)	0.59
Nurse at home, n (%)	14 (13.7)	3 (7.3)	6 (19.4)	5 (16.7)	0.35
Living in residential care centre, n (%)	2 (2.0)	3 (7.3)	1 (3.2)	1 (3.3)	0.52
Higher education (bachelor/master), n (%)	21 (20.6)	7 (17.1)	7 (22.6)	7 (23.3)	0.55
**Comorbidities**					
Diabetes	19 (18.6)	9 (22)	3 (9.7)	7 (23.3)	0.33
Asthma	6 (5.9)	2 (4.9)	2 (6.5)	2 (6.7)	0.93
COPD	4 (3.9)	3 (7.3)	1 (3.2)	0 (0)	0.30
Parkinson’s	3 (2.9)	0 (0)	2 (6.5)	1 (3.3)	0.27
Prolapse	60 (58.8)	27 (65.9)	15 (48.4)	18 (60)	0.46
**Clinical Parameters**					
Blood pressure systolic, mmHg (median, IQR)	135 (122–150)	137.5 (120.25–156)	136 (125–150)	129.5 (120–144.75)	0.38
Blood pressure diastolic, mmHg (median, IQR)	74 (69–80)	76 (71–80.75)	74 (66–83)	73.5 (69–77.75)	0.40
Length, meter (median, IQR)	1.62 (1.56–1.67)	1.62 (1.55–1.67)	1.65 (1.58–1.68)	1,61 (1.53–1.67)	0.36
Weight, kg (median, IQR)	70 (62–80.75)	69 (58.5–81.25)	70 (62–80.5)	71.9 (62.75–82)	0.64

IQR: Interquartile range, SUI: Stress urinary incontinence, UUI: Urgency urinary incontinence, MUI: Mixed urinary incontinence.

**Table 2:** Urodynamic parameters in different positions.

Urodynamic Parameter, Median (IQR)	Sitting vs. Standing, General Study Population (n = 102)	Sitting vs. Standing, SUI (n = 41)	Sitting vs. Standing, UUI (n = 31)	Sitting vs. Standing, MUI (n = 30)
**First sensation of bladder filling, mL**	185 (119.8–246.8) vs. 202.5 (122.8–280)	206 (122.5–288) vs. 209.5 (152.5–314.3)	134 (104–224) vs. 160 (58–280)	190 (135–255.5) vs. 210 (121–262)
**Normal desire to void, mL**	238 (188.8–327) vs. 238 (157.5–306)	240.5 (189.5–371.8) vs. 250 (181–342)	207.5 (147.5–280.5) vs. 202 (117.5–280.5)	247.5 (205–334.8) vs. 245 (154–208.5)
**Strong desire to void, mL**	340 (281.3–417.8) vs. 312 (270–407)	344 (297.8–427) vs. 340 (286.3–405.8)	270 (223.5–372.5) vs. 280 (214.5–428)	355 (300–428) vs. 300 (251.8–405.3)
**Maximum flow, mL/s**	14.7 (8.2–22.1) vs. 15.2 (10.3–22.8)	17.7 (9.2–24.8) vs. 20.3 (13.5–24.6)	11.7 (4.9–15.9) vs. 11.6 (7.3–21.2)	14.2 (9.3–21.9- vs. 12.1 (8.1–18.7)
**Average flow rate, mL/s**	3.8 (2.5–6.3) vs. 4.7 (2.7–6.5)	4.8 (2–7.6) vs. 5.6 (3.2–7.8)	2.9 (1.9–4.2) vs. 3.6 (2.3–5.9)	3.6 (2.7–6.3) vs. 3.6 (2.0–6.4)
**Flow time, s**	6.8 (4.9–11.2) vs. 6.3 (4.6–8.8)	7.4 (5.3–11.7) vs. 6.4 (5.3–8.6)	5.6 (4.4–11.6) vs. 4.8 (3.3–8.2)	7.4 (5.3–11.1) vs. 7.5 (4.3–9.9)
**Time to maximum urinary flow rate, s**	2.7 (1.6–7.5) vs. 2.0 (1.2–4.3)	3.6 (1.8–9.4) vs. 2.6 (1.7–4.5)	2.0 (1.3–11.2) vs. 1.5 (0.8–3.1)	2.7 (1.4–4.1) vs. 1.9 (1.1–5.5)
**Voided volume, mL**	279 (147.2–399.7) vs. 260 (183.7–371)	326.5 (230.1–431.5) vs. 309.1 (246.9–406.9)	191.4 (127.8–282.3) vs. 215.4 (124.1–292.5)	320.6 (143.3–440.7) vs. 250 (161.3–338.6)
**Pressure at maximum flow, cmH_2_O**	18.5 (8.8–31) vs. 16.7 (6.5–30.2)	18.3 (9.4–25.9) vs. 14.7 (4.0–34.8)	23.3 (17.4–33.3) vs. 18.1 (5.3–26.0)	11.7 (4.9–33) vs. 18.7 (8.7–26.1)
**Peak pressure, cmH_2_O**	32.8 (20.5–48.1) vs. 28.7 (16.6–47.5)	29.2 (17.4–43.1) vs. 27.5 (14.1–46.2)	42.3 (26.8–55.7) vs. 8.0 (17.6–52.7)	29.5 (18.7–47.7) vs. 31.4 (22.1–49.6)
**Mean pressure, cmH_2_O**	21 (10.1–34.8) vs. 15 (6.8–27.1)	17.1 (10.2–27.4) vs. 9.7 (4.3–24.6)	28.3 (20.4–45.3) vs. 18.9 (8.5–31.9)	17.1 (7.4–31.2) vs. 18.6 (7.3–26.4)
**Post void residual volume, mL**	**50 (0–180) vs. 40 (0–114)** ***p* = 0.026**	31 (0–156) vs. 0 (0–80)	65.5 (0.5–159.8) vs. 40 (2.4–117.5)	52 (0–230) vs. 80 (0–130)
**Compliance, mL/cmH_2_O**	40.5 (23.2–77.2) vs. 44.8 (21.6–121)	**47.7 (32.1–97.0) vs. 94.0 (39.4–240.5) *p* = 0.026**	26.4 (18.4–71.3) vs. 27 (9.3–75.5)	37.7 (21.5–97.3) vs. 31.0 (18.4–73.0)

Significant: Bold, IQR: Interquartile range, SUI: Stress urinary incontinence, UUI: Urgency urinary incontinence, MUI: Mixed urinary incontinence, mL: milliliter, s: seconds, cm: centimeter.

## Data Availability

The data presented in this study are available on request from the corresponding author.
